# The Experiences of Women who Live with Pelvic Floor Disorders: A Qualitative Study

**DOI:** 10.30476/ijcbnm.2021.87275.1422

**Published:** 2021-04

**Authors:** Zahra Hadizadeh-Talasaz, Talat Khadivzadeh, Hossein Ebrahimipour, Nayereh Khadem Ghaebi

**Affiliations:** 1 Student Research Committee, Mashhad University of Medical Sciences, Mashhad, Iran; 2 Department of Midwifery, School of Nursing and Midwifery, Mashhad University of Medical Sciences, Mashhad, Iran; 3 Social Determinants of Health Research Center, Mashhad University of Medical Sciences, Mashhad, Iran; 4 Department of Obstetrics and Gynecology, School of Medicine, Mashhad University of Medical Sciences, Mashhad, Iran

**Keywords:** Pelvic organ prolapse, Pelvic floor disorders, QualitativeResearch, Self-management, Women

## Abstract

**Background:**

Pelvic floor disorders (PFDs) are common and complicated problems that occur in women with different ages and cultural backgrounds and affect various dimensions of their life. Because of the dearth of information about how the Iranian women manage these disorders, this study was conducted to explore the experiences of women who live with PFDs.

**Methods:**

This qualitative study was conducted between 2018 -2019 on women who referred to the clinics of Mashhad educational hospitals . 25 deep and semi-structured interview with 22 patients with PFDs was done. They were recruited through a purposive sampling method among women with diagnosis of PFDs. Data were analyzed using conventional content analysis adopted by Graneheim and Lundman and organized using the MAXQDA software (Ver.10).

**Results:**

The analysis of the data led to the emergence of a theme of “Acceptance and Tolerance”, including four categories: “Trying to adopt sexual conflicts”, “ Concealing the disease”, “Trying to modify the lifestyle”, and “Controlling negative emotions”, and 15 sub-categories.

**Conclusion:**

This study provides an insight into self-management strategies for different aspects of challenges faced by women with PFDs.They try to resolve, conceal, modify, and control some issues to accept and tolerate their disease. By identifying self-management strategies, care providers can design and implement counseling, educating and supporting interventions, and also a program through which the patients help and guide each other.

## INTRODUCTION

Pelvic floor disorders (PFDs) such as urine and fecal incontinence and pelvic organ prolapse are stressful, annoying,chronic conditions that affect the life of millions of women of all ages in the world and lead to considerable impairments in the quality of life. ^1^

Studies have indicated that 11.5 to 35 percent of women suffer from PFDs worldwide. ^2 - 4^ The prevalence varies around the world. The results of a study in United States predicted that by 2030, request for care for PFDs will have increased about 35%. ^5^ One study declared that already one in five women in Ethiopia had PFDs. ^2^ A cross-sectional study in Babol, Iran, on urinary tract disorders due to pelvic floor disorders showed that stress incontinence was seen in 45%, urgent incontinence in 22.5% and mixed type in 32% of women above 18y/o. ^6^ Another study in Ilam city, regardless of the specific age range, reported 80% prevalence of pelvic organ prolapse. ^7^

It is generally expected that PFDs have so many impacts on different aspects of women’s daily life. ^8^ Women with PFDs may experience numerous personal and interpersonal challenges such as sexual difficulties, social problem, psychological and functional impairment, skin infections, ulcer, anxiety and depression, lack of confidence, guilt, inability to perform religious duties, sleep problems, and isolation. All of these problems can affect their quality of life. ^9 , 10^ Although those affected with PFDs experience numerous challenges, only a few of them seek professional help. Analysis of health statistics shows that women are more likely to seek treatment for their health problems and illnesses, but about the genitourinary system, women show less help-seeking behaviors. This happens due to shame, fear of invasive treatment, and untreatability; the other reasons are they believe that the symptoms belong to the natural aging process or think about it as a manageable condition. ^11 - 13^

PFDs are regarded as a chronic condition that needs long-term management. ^14^ In other studies, self-management is frequently used for other chronic conditions such asarthritis, asthma or diabetes and it has been shown to be effective and promote the people’s quality of life. ^15 , 16^ Self-management is described as the individual’s ability to manage not only the physical symptoms, but also the emotional and social consequences of a condition. ^17^ Self-management is a dynamic and active process with aspects of learning, trial and error, and exploration of limitations. ^13^ Management of chronic conditions is important to ameliorate daily problems, and also improve the quality of life. ^18^ Management strategies are instigated by the woman herself or suggested by their clinician. ^13^ A review of studies shows that few studies have been conducted on self-management of patients with PFDs in qualitative approach. ^11 , 13^ One study investigated coping styles of the elderly women with stress urinary incontinence in a cross-sectional survey. ^19^ Another study determined medical and self-care practices with quantitative design. ^20^ Step by step stages of qualitative studies can achieve a deeper understanding of self-management strategies. A qualitative study declared that personality and culture had a significant impact on management strategies. ^21^ A review of the previous studies conducted in Iran showed that there was no qualitative study on this subject. According to the purpose and question of the research, content analysis method is suitable for this study.This method has felexibility and extracts the research concepts needed from the text data in the simplest way. ^22^ In order to improve the health care provision for PFDs women in Iran, a qualitative research is necessary to show how women respond to and manage health threats in daily life. Therefore, this study was conducted to provide a deep understanding of the women’s experiences who live with PFDs.

## MATERIALS AND METHODS

In this research, a qualitative conventional content analysis was conducted from March 2018 to June 2019. Conventional content analysis is used if the purpose of the research question is to reach a new and general perspective, to introduce reality, and ultimately to create concepts that describe the phenomenon. Also, a research article or theory existing on a phenomenon is limited. In this case, researchers avoid using predefined categories. ^22^

The study population consisted of women with any kind of symptomatic PFDs who referred to the gynecology clinics in several academic hospitals affiliated to Mashhad University of Medical Sciences, Mashhad, Iran. The inclusion criteria for our study consisted of patients with chief complaints of pelvic organ prolapse (POP) of any compartment at any stage or any types of urinary incontinence (UI) or bowel dysfunction and age older than 18 years. Diagnoses were confirmed by the first researcher and physician based on the history and physical examinationin dorsal lithotomy position. The exclusion criteria of the study were being pregnant or in postpartum period, and having dementia. Purposive sampling was used to include a range of characteristics that might reflect diversity, i.e. age, education, job, socioeconomic status, type of delivery, gravidity, duration of having disorder, and type of pelvic disorder. Finally, 22 participants were enrolled in this study.

Data were collected during 16 months. In order to gain an insight into the experiences of women with PFDs, we performed individual, face-to-face semi-structured interviews. All interviews were conducted by the first author, who had passed courses on qualitative approaches, had participated in several qualitative workshops, and had been trained in interviewing techniques. For gathering the data during interview sessions, an interview guide was provided by reviewing the related literature and consulting with experts.The interview started with general question such as “Could you describe the daily experience of living with pelvic floor disorders?”; then, the participants were asked more detailed questions as the interview advanced such as “What strategies have you used to deal with body changes and its problems?” and “Describe your experience of daily self-management”. Also, they were asked probing questions to get additional information such as “Could you tell me more, please?”, “Could you give an example, please?” and “What did you mean when you tell……?”. All interviews were done in the Persian language; then, they were translated into the English language. Data collection continued until we reached data saturation and no new category emerged from the data. The interviews were done at a time and place that was suitable for the participants. Overall, most interviews were conducted at the hospital, two at participants’ in their houses, and two at the participants’ workplaces. The interview duration was between 20-65 minutes. All interviews were digitally recorded and transcribed verbatim for analysis.

Data collection and analysis were conducted concurrently. All interviews were entered and managed into MAXQDA software (version 10, VERBI Software, Berlin, Germany). Data analysis was done using conventional content analysis based on Graneheim and Lundman’s approach (2004). ^23^ After transcription, each interview was read several times in order to get an idea of the whole contents and immerse into the raw data. Then, meaning units (e.g. words, sentences, and paragraphs) were identified and abstracted and given a descriptive code. In the next step, based on the similarities and differences, the meaning units were organized into subcategories, and then similar subcategories were placed in the category. Finally, categories developed to themes.

In this study, Lincoln and Guba’s (1985) proposed criteria were used to ensure the trustworthiness and rigor of qualitative research findings, including credibility, transferability, confirmability, and dependability. In this study, credibility of data was obtained through prolonged engagement (about 14 month) for collecting and analyzing the data. Furthermore, variation was considered in selecting the participants to access a variety of experiences. Also, the supervisors (the second, the third and fourth author) checked the processes of interviewing, coding, categorizing, and interpreting the findings. In the member check, the coded interviews were given to three participants to get an agreement between the researchers and participants. For transferability, a broad and clear description of demographic characteristics of the participants and the study context was provided to enable the reader to decide about using the results. To ensure dependability, the researcher gave data to three outside observes who were not in the research team, but they were familiar with PFDs and qualitative studies to examin and confirm the data. For confirmability, three reviewers who were not in the research team reviewed the findings, interpretations, and conclusions of the study. Also, using suitable quotations in the findings can help confirmability.

Ethics approval was obtained from the Ethics Committee of Mashhad University of Medical Sciences in Iran (Code of EthicsIR.MUMS.REC.1396.365). Prior to the start of the interviews, the participants were informed about the purpose of the study and signed written consent. Participants were assured that their information would be kept confidential and the interview audio files would be kept in a safe place.

## RESULTS

In this study, 25 interviews were done with 22 participants. Three women were interviewed for more than once in order to achieve more information and better understanding of the concepts obtained and fill the gaps identified during the analysis. The demographic characteristics of the participants are presented in Table 1. The participants’ mean age was 45.05±10.52 years (range 28-65).

**Table 1:** Characteristics of the participants included in the study

Participant	Age (year)	Education	Occupation	Type of delivery	Gravidity	Menopause status	Duration of having disorder (years)	POP-Q Stage*
1	49	High school (9-12)	Carpet weaving	NVD &CS	4	Postmenopausal	14	Not observed
2	65	Secondary (6-8)	Housewife	NVD	4	Postmenopausal	8	II
3	35	Secondary (6-8)	Housewife	NVD &CS	4	Premenopausal	9	II
4	28	Secondary (6-8)	Carpet weaving	NVD	2	Premenopausal	5	III
5	43	Secondary (6-8)	Housewife	NVD	4	Postmenopausal	9	III
6	54	High school (9-12)	Sports coach	CS	4	Postmenopausal	15	I
7	36	High school (9-12)	Housewife	NVD	4	Premenopausal	2	II
8	58	Primary (1-5)	bakery	NVD	6	Postmenopausal	6	II
9	49	University (13+)	Hair dresser	NVD	3	Premenopausal	7	II
10	35	High school (9-12)	Secretary	NVD	2	Premenopausal	3	Not observed
11	38	Primary (1-5)	Housewife	NVD	6	Premenopausal	7	III
12	43	Primary (1-5)	Farmer	NVD &CS	5	Premenopausal	4	III
13	45	Guidance (6-8)	Housewife	CS	1	Premenopausal	4	II
14	32	High school (9-12)	Teacher	NVD	3	Premenopausal	3	II
15	48	Primary (1-5)	Housewife	NVD	5	Postmenopausal	1	II
16	61	Secondary (6-8)	Housewife	NVD &CS	7	Postmenopausal	7	Not observed
17	50	Secondary (6-8)	home worker	NVD	4	Postmenopausal	7	IV
18	55	High school (9-12)	Chef	NVD	2	Postmenopausal	2	II
19	38	Primary (1-5)	home worker	NVD &CS	3	Premenopausal	5	II
20	30	University (13+)	Housewife	NVD	2	Premenopausal	5	II
21	41	High school (9-12)	Housewife	NVD &CS	3	Premenopausal	6	II
22	58	Primary (1-5)	Farmer	-	0	Postmenopausal	8	II

* Pelvic Organ Prolapse Quantification (POP-Q) Stage

In the present study, 15 subcategories, four categories, and one theme emerged from the data analysis, which are discussed below (Table 2). The emerged theme was *“Acceptance and Tolerance”* captured from the experiences of the women living with PFDs. By passage of time, the participants in the face of the symptoms of PFDs tried to accept the disease and concerns arising from it as a part of their lives and make it more tolerable by doing some strategies. These strategies include trying to resolve sexual conflicts, concealing the disease, trying to modify lifestyle, and controlling negative emotions.

**Table 2:** Subcategories, categories, and themes

Subcategories	Categories	Theme
Adopting sex without pleasure	Trying to adopt sexual conflicts	Acceptance and Tolerance
Trying to alleviate problems in sex		
Adopting compatible and incompatible approaches to sexual violence		
Reducing social interactions and activities	Concealing the disease	
Taking individual considerations to conceal the disease		
Conscious choice in disclosure		
Promoting and maintaining physical strength and fitness	Trying to modify lifestyle	
Matching nutrition tailored to individual circumstances		
Moderating everyday affairs		
Probing information from various sources actively		
Avoiding stressful environments	Controlling negative emotions	
Hoping for divine healing		
Neglecting the problem		
Doing some fun domestic activities		
Seeking social support		

### 1. Trying to Adopt Sexual Conflicts

Trying to adopt sexual conflicts was one of the strategies used by the participants.The category consists of three sub-categories: *“Adopting sex without pleasure”*, *“Trying to alleviate problems in sex”*, *“Adopting compatible and incompatible approaches to sexual violence”*.

### 1.a. Adopting Sex Without Pleasure

Due to numerous physical and mental problems in women with PFD, such as pain and decreased libido, the participants did not enjoy their relationship well and had sex with reluctance. Maintaining their cohabitation and family foundation, having strong religious beliefs about satisfying the men’s needs and gaining peace of mind that the husband does not betray on them were extracted from the participants’ interview about adopting sex without pleasure. A young woman with religious beliefs described: *“I have no desire and no pleasure, but it is necessary that the man’s needs be met; otherwise, God will be angry. I have pain and I don’t want to, but whenever my husband needs me, I do not refuse. I know God will give me his reward and will be pleased with me because I have not made a man be sinful”* (P14, 32 y/o)

Another participant who worried that her husband would not betray her, said: *“In sex, I express my feelings in a way that I feel like I am enjoying I always pretend, but actualy I don’t enjoy”* (P20, 30 y/o)

### 1.b. Trying to Alleviate Problems in Sex

Most participants stated that sexual problems were one of the most important interpersonal concerns and they tried to make it more tolerable. Most participants tried to choose the right position that is less likely to cause discomfort. Using lubricant, herbal medicine, and squeezing the pelvic muscles were the other strategies to alleviate problems in sex. *“I don’t feel anything, I just feel my vagina becomes so wide; I try to squeeze muscles during intercourse; this style is a little helpful for me ”* (P20, 30 y/o) Another participant said: *“Because of low libido and pain, I use lubricant. This way reduces the pain for me.”* (P3, 35 y/o)

### 1.c. Adopting Compatible and Incompatible Approaches to Sexual Violence

Most women stated that they had experienced some form of oppressive sex with their husbands, and in response to such sex, they used a variety of strategies from compatible to incompatible depending on their personal lives, their personality charactristics, and depth of emotional relationship with their spouse.

Most women with compatible approaches stated that culturally and socially satisfaying men is an important thing that they must have self-devotion and tolerate.*“Sometimes I forget that I have husband at all. We don’t talk to each other. His behavior has become normal for me. Until now I have endured and sacrificed myself”* (P8, 58 y/o)

Some participants accepted their husbands’ behavior that was unpleasant, by thinking of his good traits. *“Although I say I am sick, he wants sex. He’s not a bad man. He is a hard-working man and loves his children and family, but in this issue, he does not pay attention. I have accepted him like this”* (P1, 49 y/o)

A number of women with incompatible approaches responded to men’s violence by adopting negative emotional and psychological revenge.*“We’re fighting a lot now ... Some nights, I sleep at my son’s house and leave him alone, so I deprive him of sex”* (P17, 50 y/o)

Some participants cursed their husbands because they did not support them. *“He lives for himself and I do for myself. I don’t talk with him. I wish that God would make him sick and no one would give him a hand”*(P15, 48 y/o)

### 2. Concealing the Disease

Most participants with PFD demonstrated actions that showed they tried to hide their problems. Negative attitudes toward PFD, fear of being ridiculed, stigma caused by the illness, and embarrassing nature of the illness caused decreased self-esteem and shame if people were informed about the disease. For the mentioned reasons, people prefer to hide their illness and not disclose their disease as much as possible and only disclose it under certain circumstances. Concealing the disease category consisted of three subcategories of reduction of social interactions and activities, individual considerations to conceal the disease, and conscious choice in disclosure.

### 2.a. Reduction of Social Interactions and Activities

Most participants stated that they always felt worried lest their friends, relatives or other people know about their problem. Therefore, they did not want to attend the friends and acquaintances’ ceremonies, religious places, travel long distances, and take part insocial gatherings. *“I’m not going to the holy places because I know I will get dirty with even sneezing or coughing. Then, no more praying, no pilgrimage”* (P16, 61 y/o)

Some working women stated that they had to quit or relocate their job because of illness problems such as pain, loss of energy, feeling pressure, urine incontinence and all physical problems caused by PFD. *“I try to go out less or go to places where I know there is a toilet. I also have a carpet weaving workshop, but I closed it. Since then I have been working in the house because I had to have some rests during my work”* (P1, 49 y/o)

### 2.b. Taking Individual Considerations to Conceal the Disease

The unpredictable nature of the symptoms causes the participants to use methods to manage them outdoors because they did not know when or where the symptoms may occur. They talked about using a variety of strategies to conceal the disease, including being always ready, carrying extra clothing, using dark clothes, not drinking liquids and considering the accessibility to toilets. For example, one of the participants described: *“Often, I wear black pants, even if my urine leaks a bit, it won’t be seen and no one will understand”* (P9, 49 y/o)

### 2.c. Conscious Choice in Disclosure

Selective disclosure was another strategy to conceal the disease. The criteria for target selection were often related to kinship, intimacy, supportive personality, and professional information. 

Most participants discussed their problem to their first-degree relatives. *“I didn’t tell anyone; only my mom and husband know about it. This is a very personal matter and it is related to the private part of your body. I don’t like others to interfere in my private life”* (P3, 35 y/o)

Especially, almost all of participants expressed their problem to their husband,. *“I talked to my mother briefly about this issue; I am not very comfortable talking to her about sexual and genital issues. I know these are personal and private matters that only concern you and your husband”*(P20, 30 y/o)

Some participants stated that they only talked to kind people, understood them, and empathized with them. For example: *“I talked to my mother-in-law. She always guides me very well; she loves me and she is always with me. Now, she is following my problems and insists on visiting to the doctor”*(P4, 28 y/o)

A number of participants believed that the best option for disclosing the problem, especially about genital and private issues, is the medical team. *“I often read or search in the Internet, and ask my doctor; these are more reliable sources. I restrict my privacy”* (P20, 30 y/o)

### 3. Trying to Modify the Lifestyle

The majority of participants declared that they tried to cope with their situations by modifying their lifestyle based on individual circumstances.These strategies acted as a moderator between the stressors and disease. These strategies were sometimes suggested by the medical team; often, women found them in searching the resources or inquiries from other people. Also, they had acquired them by themselves through their life experiences when managing the symptoms of their illness. This category consisted of four subcategories of promoting and maintaining physical strength and fitness, matching nutrition tailored to individual circumstances, moderating everyday affairs, and actively probing information from various sources.

### 3.a. Promoting and Maintaining Physical Strength and Fitness

Women realized the benefits of physical exercise to improve their condition by recommending others or searching in different sources. Therefore, they persuaded themselves to do so. Doing Kegel exercise, losing weight, walking in the pool, and using the Jacuzzi to relieve the pain were different types of physical exercise which the participants tried to do. One of the participants who had lost weight based on the doctor’s recommendation stated: *“A month and half ago, I visited a doctor who told me to go on diet. During this period, I have lost 3-4 kilos. My incontinence improves. Before my weight loss, I used to go to the toilet 15 times a day, but now I go to the toilet 9 times a day. I hope it improved more by losing more weight because I am still overweight”* (P18, 55 y/o)

Another participant who was advised to do kegel exercise by her coach stated:*“I forced myself to do pelvic floor exercise everyday.”*(P20, 30 y/o)

### 3.b. Matching Nutrition Tailored to Individual Circumstances

Most participants found that for being able to cope with their illness and experience a partially normal life, they had to change and modify their diet. They gradually detected the diets that would aggravate or improve their conditions related to management of their symptoms; then, they adjusted their nutritional status tailored to individual circumstances. Reducing caffeine and sugar intake, adjusting their ﬂuid intake and the timing of diuretic drugs with the time of leaving home, doing sex, and going to ceremonies are some strategies that they used in order to manage their symptoms. . One of the participants with hypertension said, *“The furosemide tablet that I eat for my blood pressure makes me urinate more. I don’t eat that pill before leaving home”* (P2, 65 y/o) 

Another participant said: *“I used to drink tea 8-9 times a day; it makes me feel like going to toilet more, so I’d rather not drink it, or maybe I drink some if I don’t intend to leave home”* (P16, 61 y/o)

### 3.c. Moderating Everyday Affairs

All participants were doing some strategies to cope better with symptoms during the day. Resting more and using assistive devices (different kinds of sanitary pads, waterproof protectors for mattresses, and using soft pillow for sitting) are declared. *“I used to squat for hours for making dough and bread and lift heavy equipment. After quitting those kinds of affairs, I gradually feel better. Recently, I rest more during the day.”* (P8, 58 y/o)

Timing of voiding like before going out or before ablution, warming feet or waist, and crossing legs while sneezing or coughing are some strategies that were stated by some participants. *“I always wear socks to keep my feet warm and also I wrap my waist with a cloth. These actions caused less urinatation.”* (P16, 61 y/o)

### 3.d. Probing Information from Various Sources Actively

One of the important strategies of participants in relieving symptoms or finding the right treatment was to find out more information about PFD. Some participants believe that health professionals do not spend enough time for providing the necessary information and training. For this reason, they actively sought information and obtained it from a variety of sources including family elders, friends, peer experiences, the Internet, and books. *“I am used to searching about many things. For my PFD problem, I also searched on the Internet and found some information about the cause of PFD, different treatments, and coping strategies.”* (P20, 30 y/o)

Another participant stated: *“My niece is a nurse; I ask her about any problems or questions I have”* (P13, 45 y/o)

### 4. Controlling Negative Emotions

The majority of participants stated that they were psychologically disturbed bythe presence of their incontinence or prolapse, but some have tried to control and minimize these effects. Each person reacted differently to their negative emotions:avoiding stressful environments, hoping for divine healing, neglecting the problem, doing some fun domestic activities, and seeking social support. 

### 4.a. Avoiding Stressful Environments

Most participants stated that reduction in going outdoors and social activities relieved their stress. *“When I wantto go out, I get so stressed out about what to do with my urine, so stressed out for not messing up somewhere. Thus, I’d rather not to go”* (P2, 65 y/o)

### 4.b. Hoping for Divine Healing

Some participants stated that they hoped for divine healing and divine help that was due to their religious beliefes. They resorted to prayer, vowing, and reciting the Qur’an. *“Life, death and health are from God. I vowed and prayed a lot. If He wants, I will receive cure from him. If not,God might want me to endure this pain for lessening my sins.”* (P21, 41 y/o)

### 4.c. Negligence of the Problem

Some participants tried not to highlight their problems, and not make their situations harder by thinking a lot about the symptoms, by washing and changing their clothes constantly. *“I try not to think a lot about my problems along a day; even if I have leak, I don’t think of it, and don’t change my clothes. I just change it and wash my genital whenever I want to say my prayers.”* (P2, 65 y/o)

People tried not to magnify the disease by making it less important than other diseases, and said that, in this way they would cope with and manage their disease better. A participant declared: *“... I thought my other illnesses were more important and I did not pay much attention to my prolapse”*. (P18, 55 y/o)

### 4.d. Doing Some Fun Domestic Activities

Some women did some amusement activities at home to reduce the depression caused by the reduction in social interactions and social activities.*“Because I didn’t go out much, I got depressed. I tried to amuse myself at home, learned knitting from my daughter. By doing fun activities, I was thinking less about my illness.”* (P3, 35 y/o)

### 4.e. Seeking Social Support

Some participants prefer to express their feelings and not to suppress them.They communicate with people they trust in. They believe that social support can improve their mental disorders, help them tolerate their problems more easily and relieve their stress. Those who refused to talk to others deprived themselves from social support and could not manage their negative emotions well. *“If I have any problems, at first I talk to my husband; he cares about me and my health a lot. After that, I talk to my mother-in-law; she is so kind, sympathizes with me, and guides me about how to manage it.”* (P4, 28 y/o)

Asking support from family members was the first and most important source of support. One participant explained: *“I told my sister about my problems and asked for help. She came from Tehran to Neyshaboor and brought me to Mashhad to visit the best specialist to see what my problem is and what is its treatment.”* (P15, 48 y/o)

## DISCUSSION

The present study explored the experiences of the women with PFDs. The participants’ statements showed that they were trying to use strategies to accept the disease and make it more tolerable. In line with our results, most of the studies on other types of chronic diseases show that in chronic diseases people try to accept the disease as a constant companion over time and take some steps to further improve and make it more tolerable. ^24 , 25^ In line with our result, participants with different genital prolapse degree in a qualitative study stated that they had coped with their condition and accepted it. ^26^ In long-term illnesses, patients seem to learn how to manage their illness which can help acceptance. This learning can occur experimentally or through training.

In this study, self-management strategies to accept and tolerate PFD included trying to adopt sexual conflicts, concealing the disease, trying to modify the lifestyle, and controlling negative emotions.

Adopting sex without pleasure was declared by most of our participants. Sexual dissatisfaction is a chief complaint among women suffering from PFDs. ^27 - 29^ The results of a study showed that women used some self-management strategies to cope with symptoms such as maintenance of a sexual relationship, using humor and avoidance. ^21^ In contrast to the results of this study, some studies report abstaining from sexual activity. ^30^ One qualitative study revealed that the affected women used some strategies to manage their sexual life, one of which was avoiding having sex. ^31^ The reason for this difference can be related to culture and religion. Because in the religious conditions of Iranian society, it is obligatory to satisfy the sexual needs of men. Also, because of the importance of the family foundation, women try to tolerate sex even without pleasure. Sexuality and PFDs in Iran as well as in many cultures are the issues ignored by health care professionals and women are not used to asking questions, but women themselves used some ways to manage PFDs problem while having sexual intercourse although these strategies were not always successful.

According to the findings, women try to conceal their disease through reduction of social interactions and activities, taking individual considerations and conscious choice in disclosure.

Other qualitative studies also indicated that women with PFDs quitted their social, leisure and spiritual activities for a long period of time in order to hide their condition. However, this strategy increases their limitations and make them isolate; thus, it has negative effects on their quality of life. ^10 , 30^ Therefore, it is very important for health providers to examine its effects on their social life, and guide them how to manage it and eliminate the individuals’ fears by increasing their awareness. Disclosure of the problem was done by considering some issues, but most of them expressed that they share their condition with their family, especially their husbands. In contrast to our findings, a study among Muslim women of South Asia found that talking about woman’s problem, especially about incontinence, with their husband was culturally wrong and they just shared it with their female friends or other family members. ^32^ This depends on cultural differences. In Iran, it is more acceptable for women to talk to their husbands about genital issues than others. They believe this issue is something that is related to him. On the other hand, in our study, women disclosed it to their husband because they considered them as important supporters.

Participants tried to modify their lifestyle by promoting, moderating, matching or probing some issues. Similarly, modifying lifestyle has been identified by several studies. ^10 , 30 , 33^ For example, one study indicated that the affected women try to reduce ﬂuid intake, attend places with toilets accessible, are careful in choosing clothes, and stop using anti-hypertensive pills before leaving home. ^30^ Other studies reported that quitting smoking and drinking alcohol were also helpful in controlling their symptoms. ^34 - 36^ In our study, none of the participants mentioned alcohol or smoking as a way to control PFDs. Totally, modifying lifestyle is important to ameliorate the consequences of PFDs in everyday life. 

Controlling negative emotions were the most important coping strategies of women in the present study because negative emotions have great negative burden on individual, sexual and social life. Other studies have used similar strategies to control negative emotions such as neglecting, renaming or minimizing it and constantly avoiding danger. ^21 , 37^ The only difference in our study was the hope of divine healing with prayer and vows. A number of studies in Iran on the relationship between religiosity and mental health have shown the positive impact of religious beliefs on controlling negative emotions. ^38 - 40^ Hope to have God’s help in difficult conditions can make it more tolerable while facing stressful life events. Religious counseling by health providers can lessen negative emotions. Doing some fun domestic activities is a new issue that no previous studies have mentioned. Thus, this issue can also be recommended by health providers. If PFDs are diagnosed, then its assessment must be focused not only on the physical and medical aspects, but also on the psychosocial aspect. Therefore, health-providers, for instance, must ask about their experience and understand how it affects the everyday life. For example, if a person experiences isolation or the partnership suffers, consultation must focus on helping to manage that affected part of life. Therefore, support programs should be individually configured and oriented to the living environment and resources of the affected person.

One of the limitations of this study is the cultural dependence of topics such as PFDs, which are related to genitals, and women do not talk about these issues easily, especially when accompanied by sound recording. However, at the beginning of each interview, an attempt was made to gain the trust of the participants by fully explaining the purpose of the research and stating the confidentiality of the information. The qualitative approach of this study limits the generalizability of the findings. In addition, only the perspectives of the affected women in Mashhad have been used in this study that could be considered as a limitation. Therefore, it is suggested that similar research should be conducted to help complete our understanding about strategies in diverse groups of women in Iran.

## CONCLUSION

The findings showed that women with PFDs reported a variety of self-management strategies that could help them to accept and tolerate their disease. Women with PFDs faced a variety of challenges that decreased their quality of life; this is health care providers responsibility to design and implement counseling, educating, empowering and supporting interventions that are effective like a program in which the patients help and guide each other. Also, the health network or special magazines for women and family to create a culture for timely referral of women and improve the family’s understanding of their condition. It is suggested that a future study should be designed to evaluate the effectiveness of these interventions. Health providers can encourage the affected women to use strategies that are likely to be more effective. Also, health planners have to design appropriate preventive and care programs for these women. Future studies can investigate whether health-providers’ attention toward effective coping could assist women in managing PFDs’ challenges.
